# School health professionals’ understanding of culture: a scoping review

**DOI:** 10.1136/bmjopen-2025-100689

**Published:** 2025-07-25

**Authors:** Emmie Wahlström, Sara Landerdahl Stridsberg, Camilla Larsson, Jonas Stier

**Affiliations:** 1 School of Health, Care and Social Welfare, Mälardalen University, Västerås, Sweden; 2 University library, Mälardalen University, Västerås, Sweden

**Keywords:** Schools, Health Equity, Health Services, Primary Prevention, PUBLIC HEALTH, Review

## Abstract

**Abstract:**

**Introduction:**

Culture underpins social interaction between school health professionals and children. Both practice and research suggest that cultural variations, migration and intercultural interactions pose potential challenges in encounters between school health professionals and children and may relate to the health professionals’ understanding of their own culture as a factor in such encounters. Still, for the school health services (SHS), reviews collating existing research on school health professionals’ understanding of culture are lacking.

**Objectives:**

This review aims to identify, describe and analyse existing research on school health professionals’ (ie, school nurses, school social workers, school doctors and school psychologists) understanding of culture.

**Design:**

A scoping review of peer-reviewed and published scientific articles on school health professionals’ understanding of culture.

**Inclusion criteria:**

Articles published between 2013 and 2024 on culture, SHS and school nurses, school doctors, school social workers or school psychologists.

**Methods and analysis:**

Searches were conducted in October 2023 and September 2024 in 10 databases. Two reviewers independently screened the article titles, abstracts and full texts for inclusion. Extracted data were analysed using descriptive statistics and qualitative content analysis. The qualitative content analysis focused on content related to theoretical considerations, key findings and conceptualisations of culture.

**Results:**

From 1784 screened articles, 100 articles were screened in full text and 21 articles fulfilled the eligibility criteria. After identifying two additional articles through manual searches, a total of 23 articles were included in the review. The findings show that the articles primarily applied a quantitative study design, focused on school psychologists and school nurses and were conducted in the USA and Nordic-Baltic area. Self-understanding was mainly studied using validated instruments, leaving the conceptualisation of culture to the researchers. Still, only about half of all the articles described the theoretical conceptualisation of culture. Studies of intercultural interaction focused on the challenges of encountering ‘diverse’ children and raised concerns about barriers and hindrances to the encounters.

**Conclusions:**

This review shows that SHS professionals’ understanding of culture has mainly been studied within two SHS professions, within a narrow geographical sphere and without a theoretical stance on culture. Thus, more qualitative research, a clearer theoretical conceptualisation of culture and more research on SHS professionals’ practice and self-understanding are needed.

**STRENGTHS AND LIMITATIONS OF THIS STUDY**

- This review follows the methodological description of the Joanna Briggs Institute and adheres to the Preferred Reporting Items for Systematic Reviews and Meta-Analyses Extension for Scoping Reviews.
- A total of 10 databases (Scopus, PubMed, Cinahl Plus, SocIndex, Sociological Abstracts, Social Services Abstracts, APA PsycInfo, APA PsycArticles, Web of Science Core Collection, and Applied Social Sciences Index & Abstracts) were searched for relevant peer-reviewed articles by two research librarians.
- During article selection, the inter-rater reliability score was fair (kappa score: .30 and .35; proportionate agreement: .75 and .88), indicating assessment differences. However, all selection process discrepancies were discussed one article at a time and consensus was reached regarding decisions on the inclusion or exclusion of articles.
- Search terms were developed based on previous research on school health services (SHS) professionals and tested before conducting the review searches. Yet, relevant articles may have been excluded given the possibility of local variations in titles of the professionals working in the SHS.

## Introduction

 Culture is fundamental to humans and societies and underpins social interaction.^1^ It also varies profoundly between countries, organisations and groups of people—suggesting that cultures (ie, in the plural form), and cultural variation, must be accounted for, especially in times of unprecedented globalisation and migration.^2^ This is also an increasingly common assumption in health services, where health professionals are expected to interact with an increasingly diverse group of citizens.^3^ At the same time, both practice and research suggest that cultural variations, migration and intercultural interactions pose potential challenges to professionals.^3^,^6^ School health services (SHS) are no exception, as the number of migrant background children globally is significant today.^7^

SHS exists in at least 102 countries and often serves all school-attending children.^8^ SHS consists of different providers, in many countries located within the school premises. School nurses are the most common providers, followed by school doctors. Other common SHS professions include school psychologists or psychiatrists, school social workers, dentists and counsellors. The first four professions are among the most common SHS professions worldwide, and although SHS work content differs slightly between countries and professions, SHS professionals are typically involved in vaccinations, health education (sexual and reproductive health and nutrition), screening (vision, nutrition, dental, hearing and hypertension), and mental health screening and counselling.

Distributed over the professions, school nurses and school doctors mainly focus on vaccination, assessment and screening of children’s health, and health education. School social workers’ work varies more between^9^ and even within countries.^10^ Nevertheless, most school social workers encounter children and assist with problems related to poverty, attendance drop-out, motivation, decaying neighbourhoods and racism.^9^ For school psychologists, the work content of the profession also varies between and within countries^11^ and includes special education assessment, therapy, advising on behaviour problems and assisting other school professionals in various matters.^12^ Although there are differences between countries, SHS professionals share a unique feature by being located at schools and/or attending to the entire student population, regardless of the children’s health status. This suggests that SHS professionals encounter a multitude of children, with varying living conditions and health status and therefore are expected to consider culture in every encounter to provide high-quality health services.^13 14^

That said, accounting for culture in health encounters between professionals and children is nowadays stressed both in research and practice guidance in various healthcare services.^4^,^6^ According to the WHO, this is essential for providing high-quality health services, particularly to migrants.^3^ In addition, several theories and models have been developed to give guidance as to how culture can be considered in health encounters between professionals and patients.^15 16^ Existing research also points to the risks of health professionals attributing culture and cultural ‘factors’ mainly to the child or patient, whereas the health professionals’ understanding of their own culture as a factor might be overlooked.^4^ Still, for the SHS, less has been done to collate research on how school health professionals understand and conceptualise culture.

Exploring such conceptualisations of culture among SHS professionals is important as Kluckhohn and Strodtbeck, as early as 1961, pointed to the numerous definitions of culture.^17^ Still today, existing research is criticised for including a multitude of often ambiguous or conflicting definitions of culture, and for a lack of relative consensus on the meaning, operationalisation and use of culture.^18 19^ The lack of relative consensus runs the risk of making research on culture—and thereby professional encounters where it may be a factor—shallow or simplistic. There is also the risk that unreflectively and uncritically, culture is attributed to ‘certain homogeneous groups’ based on cultural characteristics, ethnicity, language, beliefs, values, traditions, etc. ^19 20^ Such attributions are seldom nothing more than stereotypes and are associated with an unwillingness or inability of certain people to conform to what is constructed as the majority norm. On the other hand, there is ample evidence that culture impacts the way people think, feel and interact. For this reason, considering culture is crucial in health encounters focusing on the children’s or families’ health-related needs.^4 6 18^ Similarly, such research suggests that SHS professionals’ understanding of culture is central to how they approach an encounter and how the children’s health needs are assessed and met. It is thus also important to understand how culture is conceptualised and studied in previous research on encounters between SHS professionals and children.

Given that culture is central, this review shows that there are research articles on SHS professionals’, teachers’, preschool teachers’ and counsellors’ encounters with children. Yet searches of existing scoping reviews within this field of research in the Cochrane Library, Epistemonikos, International HTA database, Open Science Framework, Social Care Online and PROSPERO before conducting this review found no reviews collating research on SHS professionals’ understanding of culture.

### Study purpose and review questions

This scoping review aims to identify, describe and analyse existing research on school health professionals’ (ie, school nurses, school social workers, school doctors and school psychologists) understanding of culture.

Review questions:

- How does research conceptualise school nurses’, school social workers’, school doctors’ and school psychologists’ understanding of culture?
- How does research conceptualise school nurses’, school social workers’, school doctors’ and school psychologists’ cultural self-understanding?
- What research has been conducted on intercultural interaction and communication in the context of school health professionals’ encounters with students and their families?

## Methods

The scoping review was guided by and conducted in accordance with the Joanna Briggs Institute (JBI) methodology for scoping reviews^21^,^24^ as well as the methodology described in the scoping review protocol.^25^ Reporting of this scoping review has followed Preferred Reporting Items for Systematic Reviews and Meta-Analyses (PRISMA) Extension for Scoping Reviews^26^ and the JBI template for reporting scoping reviews (https://jbi.global/scoping-review-network/resources).

### Data sources and search strategy

As studies on school health professionals are published in journals with a variety of scopes—for example, health, social work, psychology and medicine—systematic literature searches were conducted in the following databases: Scopus (https://www.scopus.com/), PubMed (https://pubmed.ncbi.nlm.nih.gov/), Cinahl Plus (EBSCOhost), SocIndex (EBSCOhost), Sociological Abstracts (ProQuest), Social Services Abstracts (ProQuest), APA PsycInfo (EBSCOhost), APA PsycArticles (EBSCOhost), Web of Science Core Collection (https://www.webofscience.com) and Applied Social Sciences Index & Abstracts (ProQuest). The search strategy is described in more detail in online supplemental file 1 and was tested in March 2023 in Scopus during the development of the scoping review protocol.^25^

The databases were initially searched in October 2023 using a combination of search terms related to the eligibility criteria of the review (participants, concept and context) including free-text terms and controlled vocabulary (ie, subject headings) (see online supplemental file 1). If databases shared subject headings, they were searched simultaneously. The search terms were combined using the Boolean operators AND and OR, and phrase searching and truncation when appropriate. To reflect the contemporary research field, searches were limited to studies published between 2013 and 2023, as a 10-year period was deemed reasonable. Initial search strategies were tested in each database and revised in relation to controlled vocabulary in each database.

**Figure 1:**
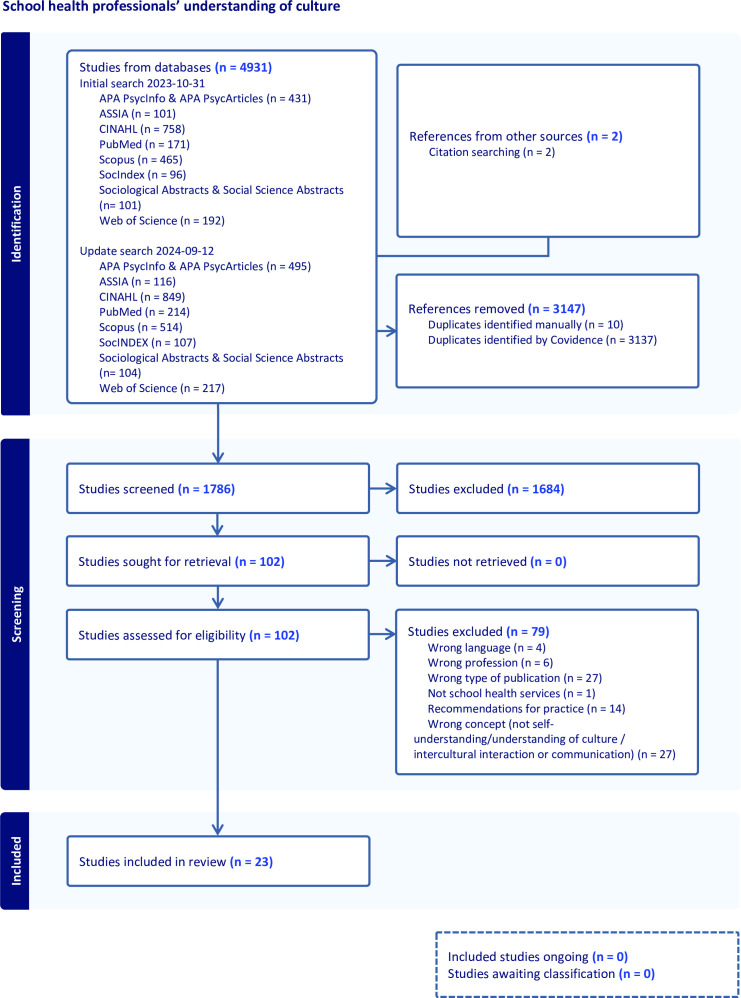
Preferred Reporting Items for Systematic Reviews and Meta-Analyses flow diagram.

An initial search was made in October 2023 and an update search was made in September 2024. No changes were made in the search syntax, or in the choice of databases. A total of 1786 references were screened. The screening process is described in a PRISMA flow diagram (figure 1). In addition, manual searches were conducted by reviewing reference lists of included studies. Relevant references cited in these studies were retrieved using Scopus and Google Scholar and read through. As a result, two articles fulfilling the eligibility criteria were included.

### Study/source of evidence selection

Covidence was used in screening (1) titles and abstracts and (2) full texts of the identified publications. The eligibility criteria of evidence selection followed the JBI guidelines on participants, concept and context.

#### Participants

Articles focusing on any of the four school health professions included in the study aim were included in the scoping review, that is, school doctors, school nurses, school psychologists and school social workers. The types of participants were included as search terms (see online supplemental file 1) with additional ‘titles’ added for school doctors and school social workers to capture the variation in how the professions are named in different geographical contexts.^25^ For an article to be included, the study participants had to work as a school doctor, school nurse, school psychologist and/or school social worker. If articles contained several professions, only data related to the relevant SHS profession were extracted. In addition, articles in which the participants were students studying to become a school doctor, school nurse, school psychologist or school social worker were excluded, given the review’s focus on school health professionals.

#### Concept

The articles’ eligibility was also assessed in relation to the studies’ key concept, that is, culture. For an article to be included, culture needed to be addressed in the title, abstract, keywords or findings. Given the research interest in investigating the conceptualisation of culture in this scoping review, all articles including the concept used or defined in any way were included, that is, any word combination where culture is a constituent part was included as a search term. Articles focusing solely on other related concepts such as social justice were excluded.

#### Context

The scoping review included articles focusing on the SHS—that is, school health professionals working at schools or regularly visiting schools where children aged 6–19 years old attend. Articles on SHS professionals at higher education institutions were not found or included. Given the fact that previous research has stressed culture as particularly relevant in encounters with migrant children, only studies on culture related to ethnicity, race or migration were included. The searches generated a few studies on culture-related encounters with LGBTQ+ children and children with hearing impairment, but these were not included in the scoping review.

#### Other criteria

Only peer-reviewed published scientific articles on either primary studies, reviews or conceptual analyses of professionals’ understanding of culture were included in the scoping review. Consequently, articles on validation of instruments or interventions, descriptions of models, editorials, debates, calls to action or similar pieces as well as books and dissertations were excluded. Another inclusion criterion pertained to language. Only texts that JS and EW were able to read (ie, in Danish, English, German, Norwegian or Swedish) were included.

#### Screening process

The screening process was conducted for the publications identified during the initial and the update search. Once all identified publications in the initial and update search had been imported into Covidence and duplicates had been removed, JS and EW screened titles and abstracts of the 1784 publications against the eligibility criteria. All publications were independently classified as ‘yes’, ‘no’ or ‘maybe’ by both reviewers. Publications assigned a ‘maybe’ or a ‘yes’ were selected for full-text screening. Full-text documents of the 100 selected publications were retrieved in full and imported into Covidence. Screening of the selected publications was conducted by the same reviewers (JS and EW) against the a priori eligibility criteria, and publications were consequently independently classified as ‘include’ or ‘exclude’. Reasons for exclusion are presented in the PRISMA flow diagram (see figure 1). Two studies that were assessed as included in the full-text screening stage were later excluded due to misalignment with the study focus. These articles were not empirical studies but descriptions of how care or counselling can account for culture. The inter-rater reliability score in the two phases was fair (kappa score: .30 and .35) and the proportionate agreement indicated that reviewers assessed the articles similarly, but with some variation (score of .75 and .88). All articles assessed differently by the reviewers at each stage were discussed between reviewers in the light of the eligibility criteria until consensus was reached. In total, the screening process and manual searches resulted in 23 articles being included in the scoping review.

### Data extraction

Data were extracted by EW from the 23 included articles using a data extraction tool developed by JS and EW (see online supplemental file 2). The data extraction was adapted from the standard instrument for data extraction available in Covidence and included author(s), title, year of publication, where the source was published or conducted (country of origin), aims/purpose, profession, study design, theoretical perspectives, population and sample size, methodology/methods, conceptualisation of culture and key findings related to the scoping review questions. During this process, extraction was continuously discussed between EW and JS.

### Data analysis and presentation

Extracted data from the 21 articles included from the database searches were exported from Covidence to Microsoft Excel (V.16.86)—organised as a spreadsheet which included all extracted details for each article sorted under each topic from the extraction tool. Extracted data from the additional two articles retrieved in the manual searches were also added to the spreadsheet. The extracted data were analysed using descriptive statistics (ie, frequency counts) and qualitative content analysis.^27^ Descriptive statistics were used to summarise, for example, the number of articles using various methodologies or conducted in certain countries. Qualitative content analysis was used to categorise data extracted within the topics: theoretical considerations, key findings and conceptualisation of culture (these are presented below under the heading ‘Foci of the literature’). Extracted texts within each topic were read through, coded and abstracted into categories based on similarities. During the analyses, relevant sections of the articles were re-read in their original context to ensure that interpretations and categorisations of extracts did not deviate from the contents of the original article. Analysis and preliminary findings were continuously discussed between JS and EW.

### Reflexivity

Given the focus of this research and the discussion on researchers’ cultural bias, it should be acknowledged that EW and JS identify as white and Swedish. EW holds a PhD in public health sciences with a focus on culture in school nurses’ encounters with children. JS holds a PhD in sociology and has extensive knowledge and experience of research and teaching in the field of intercultural interaction. SLS and CL work as research librarians at the same university as JS and EW.

## Results

In total, 23 articles on school health professionals’ understanding of culture were included in the scoping review (see online supplemental file 3). Based on these articles, the findings are organised into two sections: (1) Characteristics of the literature and (2) Foci of the literature.

### Characteristics of the literature

The included articles were published between 2014 and 2024, although most, who reported time of data collection, were conducted during 2017–2019 (n=8) (see online supplemental file 3). Studies had typically used a cross-sectional study design (n=12) and collected data using surveys (n=16). Three articles reported studies based on the same data collection.^28^,^30^

#### Population

Of the 23 included articles, most focused on school psychologists (n=12) and school nurses (n=9) (see online supplemental file 3). Two articles focused on school social workers, while no articles included school doctors. In all studies that reported gender, the majority of study participants were women (>77 %). Some articles commented on the over-representation of women, claiming that it reflected the overall population of school psychologists^31 32^ or school nurses.^29 30^ Articles reporting ethnicity or race indicated samples of mainly white,^32^,^35^ Anglo-American,^36^ European^32^ or non-Hispanic^35 37 38^ participants, with two exceptions.^31 39^ Several articles also stated that participants frequently interacted with a *specific* student population, which requires *specific* cultural considerations or sensitivity.^3034 40^,^43^

#### Concepts

About half of the articles (n=11) included descriptions of the chosen or studied framework or concept (see online supplemental file 3). In the descriptions of the chosen or studied framework or concept, the theoretical depth varied from *stating* a concept and what it contains to *explaining* the theoretical positions and constructions embedded in the concept. In articles *stating* a concept, the theoretical description was limited to a concise definition of the concept used,^29 36 42 44^ whereas those *explaining* the concept provided a lengthier description of the frameworks or models used.^2838 45^,^49^ Descriptions of frameworks and concepts focusing on culture were more common in articles on school social workers (100%; 2 of 2) whereas theoretical descriptions or clarifications were found in 42% of the articles on school psychologists (5 of 12) and 44% of the articles on school nurses (4 of 9).

#### Context

The reported studies were mainly conducted in the USA (n=13) and the Nordic-Baltic area, with five from Sweden and one from Finland^41^ and Lithuania^49^ (see online supplemental file 3). Although five articles reported studies in Sweden, three of these were based on the same data collection^28^,^30^—and all five reported studies investigating school nurses. Still, articles on school nurses were the most geographically spread (USA=2, Sweden=5, Finland=1, Korea=1). All studies on school social workers and all but three studies^40 47 49^ investigating school psychologists were conducted in the USA.

The investigated SHS professionals worked in a variation of settings, although most professionals in each publication worked in urban or suburban districts and public or government schools. Still, when comparing the articles in relation to SHS professions, no pattern emerged.

### Foci of the literature on school healthcare professionals and culture

Although the studied articles focused on various aspects related to the SHS professionals’ understanding of culture, two areas of study were particularly frequent. One area was studies exploring the professionals’ level of cultural or multicultural competence and sensitivity, or their attitudes, measured by self-reported survey data.^29 32 35 36 44 46 50^ In these studies, measurements often included both self-understanding and understanding of culture. The other area of study included descriptions or explorations of professionals’ practice, also mostly via self-reports in surveys or interviews. These studies either explored the culturally responsive, fair or cross-cultural practice of SHS professionals^31 37 38 45 49^ or investigated the influence of stereotyping on practice,^33^ or SHS professionals’ practice when encountering certain groups of students (asylum seekers,^41^ unaccompanied children,^42^ CALD students,^34 40^ children of foreign origin^30^ and newly arrived child migrants^43^). The remaining four articles had more of a theoretical focus—that is, how models and concepts can facilitate practice^39 47 48^ or test a theoretical model on cultural competence using empirical data.^28^

#### SHS professionals’ cultural or multicultural competence, sensitivity or attitudes

Overall, the SHS professionals in the articles self-reported their cultural or multicultural competence^28 29 32 35 36 46 50^ and sensitivity^44^ as moderate to high. Similarly, (multi)cultural awareness was reported as moderate to high among school psychologists^32 46^ and high among school nurses.^29 44^ In studies of school psychologists, the importance of culture was highlighted with regard to self-awareness of one’s own background and beliefs when encountering children^35^ and when developing and implementing interventions.^46^ One article suggested that implicit biases can be influential in school psychologists’ assessments of students and that cultural considerations can be lacking.^33^

Several studies indicated a relationship between higher scores in cultural domains and education on culturally related topics,^29 36 44 50^ yet two articles suggested that professionals had received little or no such training.^29 37^ Besides training or education, the articles presented relationships between scores in cultural domains and experience of encountering children ‘with other cultures’,^29 44^ personal migration experience,^29^ ethnicity,^36^ self-efficacy^44^ and geographical location of work.^36^

#### Descriptions of the SHS professionals’ practice in (inter)cultural encounters

All articles on the SHS professionals’ practice, to some extent, positioned students as needing efforts different from those of ‘mainstream’ students. Students’ status as, for example, ‘refugees’,^42^ ‘culturally and linguistically diverse’,^34 40^ ‘Latinos’^45^ or ‘Black’,^31^ suggested a need for and the importance of considering the ‘different’ culture of the student(s).^3034 38 40^,^43 45 47 49^ Professional practice was described with regard to several barriers where the lack of a shared language was mentioned frequently.^30 34 37 40 41 45^ Other barriers were the absence of material to identify cultural differences pertaining to health beliefs and practices, translated material and cultural assessment tools^37 40^ and tools generating biased assessments.^31 33^ Also, a lack of education and experience in making cultural considerations was mentioned as a problem for practice.^37 40 49^

The shortages in experience were particularly mentioned among school nurses who reported being older and having more work-life experience as beneficial for encounters with migrant children.^42 43^ For school psychologists, getting to know the ‘typicality’ of culturally and linguistically diverse students within ‘their cultural group’ was mentioned as a difficulty when making good assessments.^34 40 45^ School psychologists also reported differences in perspectives on disability between cultures as a problem in interactions with both students and families.^34^ Similarly, school nurses described that the student’s culture made certain topics sensitive to discuss (eg, sexual and mental health).^30 41^ Overall, culture was mentioned by school nurses and school psychologists as a barrier or difficulty^40 41^ and awareness of differences and cultural variations was thought to facilitate encounters.^42^

To cope with the cultural differences in norms in healthcare encounters and when discussing health topics, school nurses relied on structuring the encounter.^30 43^ During such encounters, the school nurses made recurrent adjustments to accommodate the students’ language proficiency and (assumed) culture.^30^ For school psychologists, the practice was described using other perspectives such as strength-based and assets-based approaches, which were promoted as facilitating cultural considerations and strengthening academic achievements,^45^ and a systemic or organisational perspective. The school psychologists’ role as an educator of other school personnel was described as important for strengthening other personnel’s ability to value and respect the culture of the students and for increasing the school personnel’s competence in using culturally considerate approaches with families and students.^38^ Similarly, school psychologists highlighted the importance of creating an inclusive environment, using inclusive practices, adopting a systems perspective on the encounter rather than having a within-child focus, and the importance of exploring one’s own blind spots.^47^ Similar aspects were not reported by school nurses who instead portrayed the encounters as contributing to their own learning process to become (inter)culturally competent.^42 43^

In the three articles focusing on how models and concepts can facilitate practice,^39 47 48^ a specific concept or model was suggested to facilitate intercultural interaction between SHS professionals (school social workers, or school psychologists) and children. Still, there were no similarities in the models and concepts suggested as they were based on different theoretical perspectives. Two articles presented case studies of how the model or concept could be used in practice, along with related results showing positive outcomes,^39 48^ whereas the third article presented the development of a self-reflective framework that could be used for the development of the professionals’ practice.^47^

#### Conceptualisation of culture

The articles used a variety of culture concepts, often combining or hyphenating ‘culture’ with another concept when specifying the study aims. Most common was using culture as a determiner of a noun, such as cultural *competence*^28 29 33 36 37 50^ or culturally and linguistically *diverse*.^34 40^ Culture was also combined with a prefix stating the type of culture in focus, such as *multi*cultural^35 36 39 44 46^ or *inter*cultural,^50^ but it was still used as a determiner of a noun. Used as an adjective, culture described (1) the type of practices being investigated (eg, culturally fair assessments^31^ or culturally responsive practices^46 47^), (2) something held by a professional (eg, cultural competency,^50^ cultural sensitivity^44^ or multicultural awareness^36^) or (3) a characteristic of a group of students (‘culturally and linguistically diverse’,^34 38 40^ ‘families from other cultures’^43^ or ‘culturally different student’^45^). These three types of applications of culture did not always relate to a description or definition of culture but rather used culture as an adjective that requires no further explanation and as a ‘common’ categorisation with taken-for-granted assumptions about who or what is incorporated when using the term.

 In the theory and results sections, the conceptualisation of culture illustrated a similar but slightly different pattern. Many articles conceptualised culture as being of ‘the others’, the non-members of the ‘mainstream’ or ‘typical’ population. These articles described professionals gaining or having knowledge about and/or skills to encounter ‘other’ or ‘different’ cultures simultaneously homogenising and essentialising group characteristics.^28^,^3034 37 39 41^ Culture was thus conceptualised as an individual characteristic of particular groups (having a multicultural family,^44^ coming from a cultural background^40^ or being ethnically, linguistically and racially different^32^). Similarly, culture was related to minorities^37^ and provided as a reference for understanding ‘typical’ functioning within a group.^31 33 34 49^

No studies focused solely on the professionals’ self-understanding of culture, but cultural awareness (ie, awareness of other people’s culture and/or the professional’s own culture) was investigated in a few publications.^28 29 32 46 50^ These studies focused on measuring cultural awareness, mainly using cultural competence instruments or similar scales. In the instruments, culture was mainly dichotomised between the ‘own’ culture of the professional and the ‘others’ culture’ (ie, the students’ culture). This dichotomising implies that the latter’s culture was used as a point of reference in interactions with students ‘with other’ cultures and that the students shared the same culture different from the professionals. Still, the professionals’ culture was not described in any articles. Two studies highlighted relationships between students’ race or ethnicity and culture as a reference to how culture is conceptualised.^31 33^

Some articles described intercultural interaction or communication between SHS professionals and students.^30 34 38 39 41 42 45 47 48^ All these articles contained qualitative data, gained, for example, from interviews, cases or open-ended questions. Clarifying barriers or facilitators in interactions was a reoccurring theme, but the relation to culture was not always explicit.^30 41 42 45^ Two articles described how an encounter was structured to promote ‘good’ interaction between students and professionals, and highlighted some barriers to the interaction,^30 41^ yet little was said about the cultural aspects. Similarly, another study did not mention culture as important for interaction and suggested that an open and positive attitude is sufficient and important in encounters between students and professionals.^42^ When culture was mentioned in these articles, it was often in relation to the student in negative terms—as requiring additional efforts in interactions (eg, the difference in perspectives between parents and professionals due to cultural beliefs^34^). Yet, one article described culture as a positive asset for the student but also as something necessary to consider for a certain group of students.^45^

Four articles highlighted the cultural aspects of interaction.^38 39 47 48^ In these, culture was conceptualised as shaped and re-shaped in interaction with others. Sosa *et al* illustrated cultural influence in interaction between people and between people and systems, highlighting that cultural norms embedded in societal structures and systems position migrants as a problem and that migrants need assistance in navigating among these norms.^48^ Similarly, Parker *et al* and Sakata portrayed culture as enacted at different levels in a system and as both ‘theirs’, the professionals’, and developed in interaction.^38 47^

## Discussion

Culture is studied in many disciplines and contexts. In, for example, anthropology and intercultural studies, it is even the primary concern. Yet the findings show that in research on SHS, culture is mainly used as an adjective denoting a practice, competence or a group of people, without further defining what it entails. Similar to what has been claimed for health research in general,^18^ the reviewed research, in many cases, lacks epistemological foundations, definitions or descriptions of culture, or hyphenates culture with other concepts (eg, competence or sensitivity) where culture is not defined. Additionally, conceptualisations of culture are highly diverse and often relatively shallow. Either they are very rudimentary or very precise, fixed and yet small-range definitions of culture. This gives an impression of clarity but overlooks or underconceptualises the complexity of culture. Similarly, the results in this review, and of previous research on cultural competence in health services,^18 51^ suggest that culture is often described as a fixed construct, something that you *have*, rather than something constructed and reconstructed in interaction as well as homogenous for an entire group, as a collective characteristic. In these cases, cultural dichotomisation and othering may be outcomes. Future research would, therefore, gain from using culture as a sensitising concept^52^—a middle-range, more seamless, conceptualisation guiding scientific analyses that would allow the complexity and contextuality of culture to be demonstrated.

Moreover, the findings show that there is limited research on intercultural interaction in the SHS domain—that is, on the concrete interplay of SHS professionals and children and on what de facto takes place in these encounters—and on the cultural self-understanding of SHS professionals—that is, how they express, reflect on and problematise their own cultural background. This lack of research can be contrasted with numerous frameworks and models positing how such encounters can or should be managed.^53^,^55^ This mirrors a normative idea stipulating optimal practice in intercultural encounters but also a lack of knowledge about whether this ambition is de facto enacted in practice or not. Since a lack of cultural self-understanding among healthcare professionals can result in ethnocentric attitudes and practices in health service encounters with children,^56^ knowledge of how such encounters are enacted in the SHS is vital for improving practice and counteracting discrimination.

With regard to the need for more empirical research on the intercultural interaction domain, it is also noteworthy that the findings indicate that SHS professionals’ understanding of culture has mainly been studied among two SHS professions (school psychologists n=12 and school nurses n=9). Although this review did not include the search term “educational psychologist”, indicated by Sakata^47^ to be a similar profession to the school psychologists but working in the UK, studies of school psychologists were still the most frequent. It might thus be argued that the research interest in the concept of culture may be greatest for school psychologists. At the same time, the results show that research on culture relating to school doctors seems to be non-existent. This difference in research interest between the SHS professions posits a question on why culture is more relevant to study for school psychologists and school nurses. On first glance, the answer to this question does not seem to relate to a difference between more medically oriented (school doctors and school nurses) and more psychosocially oriented (school psychologists and school social workers) professions. Nor would an answer be found if basing the explanation on professions being more or less directly accessible to students, thus differentiating between school nurses and school social workers, who in some countries are localised at school premises (eg, in Sweden^57^), and school psychologists and school doctors who in the same countries are called upon in cases where risks or problems cannot be handled by other school or SHS professionals. Thus, these questions need to be further explored by looking into the ontological and epistemological perspectives embedded in school psychologists’ and school nurses’ practices and related research while comparing them to such perspectives embedded in school social workers’ and school doctors’ practice. Still, such comparison might be difficult at the time as studies suggest a scarcity of research on these latter professions. A recent review of the characteristics of school social workers and the different types of services they deliver only identified 68 publications that were considered relevant to include after screening the title and abstract.^58^ The scarcity may be related to the diversified terminology used when describing the profession internationally.^9^ In this review, several search terms were included to ensure thorough searches, yet articles using other terminology have been left out. For school doctors, a similar argument of the need for research into school doctors’ practice can be made, although no review to validate this statement has been located. School doctors are, however, incorporated in SHS in 49 countries, suggesting that the profession is common in the SHS globally.^8^ Practices of school doctors are being studied in relation to other areas (eg, general health checks^59^), but this scoping review shows that research which incorporates school doctors and culture in any form is non-existent.

In the reviewed research, it is often unclear who has ‘operationalised’ culture—in some instances, it is the researchers, whereas in other instances, it is something that interviewees or survey respondents define. Still, in most studies, the operationalisation of culture was done by the researchers rather than by the participants, given that the studies were cross-sectional studies using a survey (validated instrument) measuring the cultural awareness, competence, sensitivity, responsiveness, etc. of participants. That said, it is interesting that so few articles offer a definition or description of culture that explains the perspective on culture chosen when conducting the research. This lack of theoretical depth on culture also highlights a lack of cultural self-reflexivity (hermeneutic preunderstanding or epic and emic approaches), which is largely absent in the reviewed research, especially in the quantitative studies. The absence of cultural self-reflexivity or at least stating the background of the researcher may lead to bias and solidification of ‘us and them’ dynamics and othering of the people or culture(s) under study.^20^ This scoping review thus calls for more qualitative research, a clearer theoretical stance regarding culture, and reflection on cultural self-reflexivity by researchers. Given the preference for quantitative methods, a related question is whether it is possible to catch perspectives and experiences related to culture using quantitative instruments or if that is too complex and therefore requires more qualitative approaches. Here, research and methodologies (including observations and ethnography) from anthropology, intercultural studies, intersectional studies and postcolonial studies can serve as inspiration. Relatedly, this scoping review also highlights a quite limited geographical distribution of studies. Included studies showed that the topic was mostly researched within the USA, although studies are found from the Nordic-Baltic region, Australia, Korea and the UK. Similarly, another review of the SHS also shows research on the SHS in general being conducted in the UK, China, Australia, Canada, Sweden and France.^8^ Thus, the area of research could be criticised for being heavily focused on the North American and to some extent western European context and, as SHS exist in 102 countries,^8^ more research in other parts of the world is needed.

Overall, research on healthcare and research would benefit from more interdisciplinary approaches, involving experts in culture research. In addition, in the reviewed research, there are also examples of frameworks that are anchored in ‘culture research’.

### Limitations

There are three main limitations to this scoping review. The inter-rater reliability in both rounds of article selection in the screening process showed a fair score, indicating that the reviewers assessed the articles differently. In the screening process, most assessment disagreements occurred in the early rounds, when the reviewers were getting acquainted to the reviewing process, the search results and the use of the eligibility criteria. During the process, discussions were held continuously on eligibility criteria and disagreements in the article selection process, thus increasing convergence in assessments. All discrepancies in the selection process were discussed one article at a time and consensus was reached regarding decisions on the inclusion or exclusion of articles. The second limitation relates to language as an inclusion criterion. In this review, only peer-reviewed articles in Danish, English, German, Norwegian and Swedish were included. During the screening phase, a few articles (n=4) were thus excluded due to being published in another language. The third limitation concerns the possibility that published articles relevant to the SHS are not identified or included. Yet search terms were developed based on previous research on SHS professionals and tested before conducting the searches in this review. In addition, given that the search results rendered many articles on school counsellors and that this profession is related to and sometimes included in the SHS, it may be of interest to conduct a separate review of school counsellors. Still, for this scoping review, school counsellors were not included as this profession has a broader focus on academic achievement and career development than mainly students’ health and development.

## Conclusion

The findings in this scoping review show that overall, research on SHS professionals’ understanding of culture is sparse. Among the conducted studies, many use a cross-sectional design to investigate the professionals’ cultural competence, awareness, sensitivity, responsiveness, and so forth, whereas there are fewer studies using qualitative methods to investigate the concrete interplay of school doctors, school nurses, school psychologists, school social workers and children. Similarly, there is also a lack of studies on how the SHS describe their cultural self-understanding. The conceptualisation of culture in published research is usually provided by researchers rather than professionals, if such a conceptualisation is described or defined. Culture is mainly used as an adjective denoting practice, competence or a group of people, without further defining what it entails, and a theoretical stance regarding culture is often missing. The findings also show that SHS professionals’ understanding of culture has been studied mainly among two SHS professions (school psychologists and school nurses) and within a narrow geographical sphere. This scoping review thus calls for more qualitative research, a clearer theoretical stance regarding culture in articles on this topic, and more research on SHS professionals internationally as well as on school social workers and school doctors, in particular.

## Supplementary material

10.1136/bmjopen-2025-100689 online supplemental file 1

10.1136/bmjopen-2025-100689 online supplemental file 2

10.1136/bmjopen-2025-100689 online supplemental file 3

## Data Availability

Data sharing not applicable as no datasets generated and/or analysed for this study.
