# Supplementary material for: School health professionals’ understanding of culture: a scoping review
Source: BMJ Open. 2025 Jul 25;15(7):e100689. doi: 10.1136/bmjopen-2025-100689 (PMC12306337; doi:10.1136/bmjopen-2025-100689)
Supplement: online supplemental file 1 [file bmjopen-15-7-s001.docx]

# Supplementary file 1 – Search tables

### PubMed (<https://pubmed.ncbi.nlm.nih.gov/>)

| **Search#** | *Exact search string* |
| --- | --- |
| 1. | "School Health Services"[Mesh:NoExp] OR "School Mental Health Services"[Mesh] OR "School Nursing"[Mesh] |
| 2. | "school health"[Title/Abstract] OR "school based health"[Title/Abstract] OR "school nurs*"[Title/Abstract] OR "school mental health service*"[Title/Abstract] OR "school based mental health service*"[Title/Abstract] OR "school social work*"[Title/Abstract] OR "school welfare officer*"[Title/Abstract] OR "education social worker"[Title/Abstract] OR "education welfare officer*"[Title/Abstract] OR "attendance counselor"[Title/Abstract] OR "school counsel*"[Title/Abstract] OR "guidance counselor*"[Title/Abstract] OR "school doctor*"[Title/Abstract] OR "school physician*"[Title/Abstract] OR "school psych*"[Title/Abstract] |
| 3. | 1 OR 2 |
| 4. | "Transcultural Nursing"[Mesh] OR "Culture"[Mesh] OR "Culturally Competent Care"[Mesh] OR "Social Identification"[Mesh] |
| 5. | "cultural* competen*"[Title/Abstract] OR "cultural* sensitiv*"[Title/Abstract] OR transcultural[Title/Abstract] OR "cross cultural"[Title/Abstract] OR "cultural* congruent*"[Title/Abstract] OR "cultural care"[Title/Abstract] OR multicultur*[Title/Abstract] OR "cultural divers*"[Title/Abstract] OR intercultural[Title/Abstract] OR "cultural awareness"[Title/Abstract] OR "cultural nursing"[Title/Abstract] OR "cultural communication"[Title/Abstract] OR "cultural skill*"[Title/Abstract] OR "cultural understanding"[Title/Abstract] OR "cultural interaction*"[Title/Abstract] OR "cultural knowledge"[Title/Abstract] OR "cultural proficienc*"[Title/Abstract] OR "cultural dynamic*"[Title/Abstract] OR "cultural safety"[Title/Abstract] OR "cultural meeting"[Title/Abstract] OR "cultural ident*"[Title/Abstract] OR "cultural self*"[Title/Abstract] OR "cultural bias*"[Title/Abstract] OR "intercultural encounter*"[Title/Abstract] OR "intercultural interaction*"[Title/Abstract] OR "intercultural exchange"[Title/Abstract] OR "ethnocultural"[Title/Abstract] |
|  | 4 OR 5 |
|  | 3 AND 6 |
| Limiters | 2013- |
| **Total** | Result numbers and search dates: **214** results on September 12, 2024  (171 results on October 31, 2023) |

### Cinahl Plus (EBSCOhost)

**(Search modes** - Find all my search terms)

| **Search#** | *Exact search string* |
| --- | --- |
| 1. | (MH "School Health Services+") |
| 2. | (TI "school health" OR AB "school health") OR (TI "school based health" OR AB "school based health") OR (TI "school nurs*" OR AB "school nurs*") OR (TI "school mental health service*" OR AB "school mental health service*") OR (TI "school based mental health service*" OR AB "school based mental health service*") OR (TI "school social work*" OR AB "school social work*") OR (TI "school welfare officer*" OR AB "school welfare officer*") OR (TI "education social worker" OR AB "education social worker") OR (TI "education welfare officer*" OR AB "education welfare officer*") OR (TI "attendance counselor" OR AB "attendance counselor") OR (TI "school counsel*" OR AB "school counsel*") OR (TI "guidance counselor*" OR AB "guidance counselor*") OR (TI "school doctor*" OR AB "school doctor*") OR (TI "school physician*" OR AB "school physician*") OR (TI "school psych*" OR AB "school psych*") |
| 3. | 1 OR 2 |
| 4. | (MH "Cultural Competence") OR (MH "Transcultural Nursing") OR (MH "Culture+") OR (MH "Transcultural Care") OR (MH "Cultural Safety") OR (MH "Social Identity") OR (MH "Cultural Sensitivity") OR (MH "Cultural Bias") |
| 5. | (TI "cultural* competen*" OR AB "cultural* competen*") OR (TI "cultural* sensitiv*" OR AB "cultural* sensitiv*") OR (TI transcultural OR AB transcultural) OR (TI "cross cultural" OR AB "cross cultural") OR (TI "cultural* congruent*" OR AB "cultural* congruent*") OR (TI "cultural care" OR AB "cultural care") OR (TI multicultur* OR AB multicultur*) OR (TI "cultural divers*" OR AB "cultural divers*") OR (TI intercultural OR AB intercultural) OR (TI "cultural awareness" OR AB "cultural awareness") OR (TI "cultural nursing" OR AB "cultural nursing") OR (TI "cultural communication" OR AB "cultural communication") OR (TI "cultural skill*" OR AB "cultural skill*") OR (TI "cultural understanding" OR AB "cultural understanding") OR (TI "cultural interaction*" OR AB "cultural interaction*") OR (TI "cultural knowledge" OR AB "cultural knowledge") OR (TI "cultural proficienc*" OR AB "cultural proficienc*") OR (TI "cultural dynamic*" OR AB "cultural dynamic*") OR (TI "cultural safety" OR AB "cultural safety") OR (TI "cultural meeting" OR AB "cultural meeting") OR (TI "cultural ident*" OR AB "cultural ident*") OR (TI "cultural self*" OR AB "cultural self*") OR (TI "cultural bias*" OR AB "cultural bias*") OR (TI "intercultural encounter*" OR AB "intercultural encounter*") OR (TI "intercultural interaction*" OR AB "intercultural interaction*") OR (TI "intercultural exchange" OR AB "intercultural exchange") OR (TI ethnocultural OR AB ethnocultural) |
| 6. | 4 OR 5 |
| 7. | 3 AND 6 |
| Limiters | 2013- |
| **Total** | Result numbers and search dates: **849** results on September 12, 2024  (758 results on October 31, 2023) |

### Social service abstract + Sociological abstracts (Proquest)

| **Search#** | *Exact search string* |
| --- | --- |
| 1. | MAINSUBJECT.EXACT("School social work") OR MAINSUBJECT.EXACT("School social workers") |
| 2. | TI,AB("school health") OR TI,AB("school based health") OR TI,AB("school nurs*") OR TI,AB("school mental health service*") OR TI,AB("school based mental health service*") OR TI,AB("school social work*") OR TI,AB("school welfare officer*") OR TI,AB("education social worker") OR TI,AB("education welfare officer*") OR TI,AB("attendance counselor") OR TI,AB("school counsel*") OR TI,AB("guidance counselor*") OR TI,AB("school doctor*") OR TI,AB("school physician*") OR TI,AB("school psych*") |
| 3. | 1 OR 2 |
| 4. | MAINSUBJECT.EXACT("Crosscultural treatment") OR MAINSUBJECT.EXACT("Cultural sensitivity") OR MAINSUBJECT.EXACT("Cultural competence") OR MAINSUBJECT.EXACT("Culture contact") OR MAINSUBJECT.EXACT("Intercultural communication") OR MAINSUBJECT.EXACT("Cultural differences") OR MAINSUBJECT.EXACT("Multiculturalism & pluralism") OR MAINSUBJECT.EXACT("Cultural Identity") |
| 5. | TI,AB("cultural* competen*") OR TI,AB("cultural* sensitiv*") OR TI,AB(transcultural) OR TI,AB("cross cultural") OR TI,AB("cultural* congruent*") OR TI,AB("cultural care") OR TI,AB(multicultur*) OR TI,AB("cultural divers*") OR TI,AB(intercultural) OR TI,AB("cultural awareness") OR TI,AB("cultural nursing") OR TI,AB("cultural communication") OR TI,AB("cultural skill*") OR TI,AB("cultural understanding") OR TI,AB("cultural interaction*") OR TI,AB("cultural knowledge") OR TI,AB("cultural proficienc*") OR TI,AB("cultural dynamic*") OR TI,AB("cultural safety") OR TI,AB("cultural meeting") OR TI,AB("cultural ident*") OR TI,AB("cultural self*") OR TI,AB("cultural bias*") OR TI,AB("intercultural encounter*") OR TI,AB("intercultural interaction*") OR TI,AB("intercultural exchange") OR TI,AB(ethnocultural) |
| 6. | 4 OR 5 |
| 7. | 3 AND 6 |
| Limiters | 2013- |
| **Total** | Result numbers and search dates: **104** results on September 12, 2024  (101 results on October 31, 2023) |

ASSIA (ProQuest)

| **Search#** | *Exact search string* |
| --- | --- |
| 1. | MAINSUBJECT.EXACT("School psychologists") OR MAINSUBJECT.EXACT("School psychology") OR MAINSUBJECT.EXACT("School nursing") OR MAINSUBJECT.EXACT("School nurses") OR MAINSUBJECT.EXACT("School social work") OR MAINSUBJECT.EXACT("School social workers") |
| 2. | TI,AB("school health") OR TI,AB("school based health") OR TI,AB("school nurs*") OR TI,AB("school mental health service*") OR TI,AB("school based mental health service*") OR TI,AB("school social work*") OR TI,AB("school welfare officer*") OR TI,AB("education social worker") OR TI,AB("education welfare officer*") OR TI,AB("attendance counselor") OR TI,AB("school counsel*") OR TI,AB("guidance counselor*") OR TI,AB("school doctor*") OR TI,AB("school physician*") OR TI,AB("school psych*") |
| 3. | 1 OR 2 |
| 4. | MAINSUBJECT.EXACT("Cultural factors") OR MAINSUBJECT.EXACT("Culture") OR MAINSUBJECT.EXACT("Cultural sensitivity") OR MAINSUBJECT.EXACT("Cultural relations") OR MAINSUBJECT.EXACT("Cultural competence") OR MAINSUBJECT.EXACT("Multiculturalism & pluralism") OR MAINSUBJECT.EXACT("Cultural competence") OR MAINSUBJECT.EXACT("Intercultural competence") OR MAINSUBJECT.EXACT("Intercultural interaction") OR MAINSUBJECT.EXACT("Intercultural sensitivity") OR MAINSUBJECT.EXACT("Intercultural communication") OR MAINSUBJECT.EXACT("Cultural Identity") OR MAINSUBJECT.EXACT("Ethnic Identity") OR MAINSUBJECT.EXACT("Ethnocentrism") |
| 5. | TI,AB("cultural* competen*") OR TI,AB("cultural* sensitiv*") OR TI,AB(transcultural) OR TI,AB("cross cultural") OR TI,AB("cultural* congruent*") OR TI,AB("cultural care") OR TI,AB(multicultur*) OR TI,AB("cultural divers*") OR TI,AB(intercultural) OR TI,AB("cultural awareness") OR TI,AB("cultural nursing") OR TI,AB("cultural communication") OR TI,AB("cultural skill*") OR TI,AB("cultural understanding") OR TI,AB("cultural interaction*") OR TI,AB("cultural knowledge") OR TI,AB("cultural proficienc*") OR TI,AB("cultural dynamic*") OR TI,AB("cultural safety") OR TI,AB("cultural meeting") OR TI,AB("cultural ident*") OR TI,AB("cultural self*") OR TI,AB("cultural bias*") OR TI,AB("intercultural encounter*") OR TI,AB("intercultural interaction*") OR TI,AB("intercultural exchange") OR TI,AB(ethnocultural) |
| 6. | 4 OR 5 |
| 7. | 3 AND 6 |
| Limiters | 2013- |
| **Total** | Result numbers and search dates: **116** results on September 12, 2024  (101 on October 31, 2023) |

### Scopus (<https://www.scopus.com/>)

| **Search#** | *Exact search string* |
| --- | --- |
| 1. | TITLE-ABS-KEY ( "school health" OR "school based health" OR "school nurs*" OR "school mental health service*" OR "school based mental health service*" OR "school social work*" OR "school welfare officer*" OR "education social worker" OR "education welfare officer*" OR "attendance counselor" OR "school counsel*" OR "guidance counselor*" OR "school doctor*" OR "school physician*" OR "school psych*") |
| 2. | TITLE-ABS-KEY ( "cultural* competen*" OR "cultural* sensitiv*" OR transcultural OR "cross cultural" OR "cultural* congruent*" OR "cultural care" OR multicultur* OR "cultural divers*" OR intercultural OR "cultural awareness" OR "cultural nursing" OR "cultural communication" OR "cultural skill*" OR "cultural understanding" OR "cultural interaction*" OR "cultural knowledge" OR "cultural proficienc*" OR "cultural dynamic*" OR "cultural safety" OR "cultural meeting" OR "cultural ident*" OR "cultural self*" OR "cultural bias*" OR "intercultural encounter*" OR "intercultural interaction*" OR "intercultural exchange" OR ethnocultural) |
| 3. | 1 AND 2 |
| Limiters | 2013- |
| **Total** | Result numbers and search dates: **514** results on September 12, 2024  (465 results on October 31, 2023) |

### Web of Science Core Collection (<http://webofscience.com/>)

| **Search#** | *Exact search string* |
| --- | --- |
| 1. | TS=("school health" OR "school based health" OR "school nurs*" OR "school mental health service*" OR "school based mental health service*" OR "school social work*" OR "school welfare officer*" OR "education social worker" OR "education welfare officer*" OR "attendance counselor" OR "school counsel*" OR "guidance counselor*" OR "school doctor*" OR "school physician*" OR "school psych*") |
| 2. | TS=("cultural* competen*" OR "cultural* sensitiv*" OR transcultural OR "cross cultural" OR "cultural* congruent*" OR "cultural care" OR multicultur* OR "cultural divers*" OR intercultural OR "cultural awareness" OR "cultural nursing" OR "cultural communication" OR "cultural skill*" OR "cultural understanding" OR "cultural interaction*" OR "cultural knowledge" OR "cultural proficienc*" OR "cultural dynamic*" OR "cultural safety" OR "cultural meeting" OR "cultural ident*" OR "cultural self*" OR "cultural bias*" OR "intercultural encounter*" OR "intercultural interaction*" OR "intercultural exchange" OR ethnocultural ) |
| 3. | 1 AND 2 |
| Limiters | 2013- |
| **Total** | Result numbers and search dates: **217** results on September 12, 2024  (192 results on October 31, 2023) |

SocINDEX (EBSCOhost)

**(Search modes** - Find all my search terms)

| **Search#** | *Exact search string* |
| --- | --- |
| 1. | DE "SCHOOL health services" OR DE "SCHOOL mental health services" |
| 2. | (TI "school health" OR AB "school health") OR (TI "school based health" OR AB "school based health") OR (TI "school nurs*" OR AB "school nurs*") OR (TI "school mental health service*" OR AB "school mental health service*") OR (TI "school based mental health service*" OR AB "school based mental health service*") OR (TI "school social work*" OR AB "school social work*") OR (TI "school welfare officer*" OR AB "school welfare officer*") OR (TI "education social worker" OR AB "education social worker") OR (TI "education welfare officer*" OR AB "education welfare officer*") OR (TI "attendance counselor" OR AB "attendance counselor") OR (TI "school counsel*" OR AB "school counsel*") OR (TI "guidance counselor*" OR AB "guidance counselor*") OR (TI "school doctor*" OR AB "school doctor*") OR (TI "school physician*" OR AB "school physician*") OR (TI "school psych*" OR AB "school psych*") |
| 3. | 1 OR 2 |
| 4. | DE "CULTURE" OR DE "CROSS-cultural communication" OR DE "TRANSCULTURAL medical care" OR DE "TRANSCULTURAL nursing" OR DE "CROSS-cultural communication" OR DE "CULTURAL competence" OR DE "CULTURAL bias" OR DE "CULTURAL Identity" OR DE "Cross-cultural studies on medical care" |
| 5. | (TI "cultural* competen*" OR AB "cultural* competen*") OR (TI "cultural* sensitiv*" OR AB "cultural* sensitiv*") OR (TI transcultural OR AB transcultural) OR (TI "cross cultural" OR AB "cross cultural") OR (TI "cultural* congruent*" OR AB "cultural* congruent*") OR (TI "cultural care" OR AB "cultural care") OR (TI multicultur* OR AB multicultur*) OR (TI "cultural divers*" OR AB "cultural divers*") OR (TI intercultural OR AB intercultural) OR (TI "cultural awareness" OR AB "cultural awareness") OR (TI "cultural nursing" OR AB "cultural nursing") OR (TI "cultural communication" OR AB "cultural communication") OR (TI "cultural skill*" OR AB "cultural skill*") OR (TI "cultural understanding" OR AB "cultural understanding") OR (TI "cultural interaction*" OR AB "cultural interaction*") OR (TI "cultural knowledge" OR AB "cultural knowledge") OR (TI "cultural proficienc*" OR AB "cultural proficienc*") OR (TI "cultural dynamic*" OR AB "cultural dynamic*") OR (TI "cultural safety" OR AB "cultural safety") OR (TI "cultural meeting" OR AB "cultural meeting") OR (TI "cultural ident*" OR AB "cultural ident*") OR (TI "cultural self*" OR AB "cultural self*") OR (TI "cultural bias*" OR AB "cultural bias*") OR (TI "intercultural encounter*" OR AB "intercultural encounter*") OR (TI "intercultural interaction*" OR AB "intercultural interaction*") OR (TI "intercultural exchange" OR AB "intercultural exchange") OR (TI ethnocultural OR AB ethnocultural) |
| 6. | 4 OR 5 |
| 7. | 3 AND 6 |
| Limiters | 2013- |
| **Total** | Result numbers and search dates: **107** results on September 12, 2024  (96 results on October 31, 2023) |

APA PsycINFO + APA PsycArticles (EBSCOhost)

**(Search modes** - Find all my search terms)

| **Search#** | *Exact search string* |
| --- | --- |
| 1. | DE "School Nurses" OR DE "School Based Mental Health Services" OR DE "School Psychologists" |
| 2. | (TI "school health" OR AB "school health") OR (TI "school based health" OR AB "school based health") OR (TI "school nurs*" OR AB "school nurs*") OR (TI "school mental health service*" OR AB "school mental health service*") OR (TI "school based mental health service*" OR AB "school based mental health service*") OR (TI "school social work*" OR AB "school social work*") OR (TI "school welfare officer*" OR AB "school welfare officer*") OR (TI "education social worker" OR AB "education social worker") OR (TI "education welfare officer*" OR AB "education welfare officer*") OR (TI "attendance counselor" OR AB "attendance counselor") OR (TI "school counsel*" OR AB "school counsel*") OR (TI "guidance counselor*" OR AB "guidance counselor*") OR (TI "school doctor*" OR AB "school doctor*") OR (TI "school physician*" OR AB "school physician*") OR (TI "school psych*" OR AB "school psych*") |
| 3. | 1 OR 2 |
| 4. | DE "Cultural Competence" OR DE "Cultural Sensitivity" OR DE "Multicultural Counseling" OR DE "Cross Cultural Counseling" OR DE "Cross Cultural Communication" OR DE "Cultural Identity" OR DE "Multiculturalism" OR DE "Cultural Bias" |
| 5. | (TI "cultural* competen*" OR AB "cultural* competen*") OR (TI "cultural* sensitiv*" OR AB "cultural* sensitiv*") OR (TI transcultural OR AB transcultural) OR (TI "cross cultural" OR AB "cross cultural") OR (TI "cultural* congruent*" OR AB "cultural* congruent*") OR (TI "cultural care" OR AB "cultural care") OR (TI multicultur* OR AB multicultur*) OR (TI "cultural divers*" OR AB "cultural divers*") OR (TI intercultural OR AB intercultural) OR (TI "cultural awareness" OR AB "cultural awareness") OR (TI "cultural nursing" OR AB "cultural nursing") OR (TI "cultural communication" OR AB "cultural communication") OR (TI "cultural skill*" OR AB "cultural skill*") OR (TI "cultural understanding" OR AB "cultural understanding") OR (TI "cultural interaction*" OR AB "cultural interaction*") OR (TI "cultural knowledge" OR AB "cultural knowledge") OR (TI "cultural proficienc*" OR AB "cultural proficienc*") OR (TI "cultural dynamic*" OR AB "cultural dynamic*") OR (TI "cultural safety" OR AB "cultural safety") OR (TI "cultural meeting" OR AB "cultural meeting") OR (TI "cultural ident*" OR AB "cultural ident*") OR (TI "cultural self*" OR AB "cultural self*") OR (TI "cultural bias*" OR AB "cultural bias*") OR (TI "intercultural encounter*" OR AB "intercultural encounter*") OR (TI "intercultural interaction*" OR AB "intercultural interaction*") OR (TI "intercultural exchange" OR AB "intercultural exchange") OR (TI ethnocultural OR AB ethnocultural) |
| 6. | 4 OR 5 |
| 7. | 3 AND 6 |
| Limiters | 2013- |
| **Total** | Result numbers and search dates: **518** results on September 12, 2024  (453 results on October 31, 2023) |
