# Supplementary material for: School health professionals’ understanding of culture: a scoping review
Source: BMJ Open. 2025 Jul 25;15(7):e100689. doi: 10.1136/bmjopen-2025-100689 (PMC12306337; doi:10.1136/bmjopen-2025-100689)
Supplement: online supplemental file 2 [file bmjopen-15-7-s002.docx]

Supplementary file 2. Data extraction instrument

| **General information** |  |  |  |  | **Methods** |  |  |  |  |  |
| --- | --- | --- | --- | --- | --- | --- | --- | --- | --- | --- |
| **Study ID** | **Title** | **Lead author contact details** | **Country in which the study was conducted** | **Year of publication** | **Aim of study** | **Profession** | **Study design** | **Theoretical perspectives (if any)** | **Start date** | **End date** |
|  |  |  |  |  |  |  |  |  |  |  |

| **Participants** |  |  |  | **Methodology** |  |  |  | **Results** |  |  |
| --- | --- | --- | --- | --- | --- | --- | --- | --- | --- | --- |
| **Population description** | **Inclusion criteria** | **Exclusion criteria** | **Total number of participants** | **Data collection 1** | **Data collection 2** | **Data analysis** | **If statistics: studied variables** | **Conceptualization of culture** | **Key findings** | **Notes** |
|  |  |  |  |  |  |  |  |  |  |  |
