# Supplementary material for: School health professionals’ understanding of culture: a scoping review
Source: BMJ Open. 2025 Jul 25;15(7):e100689. doi: 10.1136/bmjopen-2025-100689 (PMC12306337; doi:10.1136/bmjopen-2025-100689)
Supplement: online supplemental file 3 [file bmjopen-15-7-s003.docx]

Supplementary file 3 – Article characteristics

| **#** | **Author, year** | **Aim of study** | **Study design** | **Country of study** | **SHS profession** | **Theoretical conceptualization of culture** | **Period of data collection** | **Study sample** | **Data collection** | **Foci** |
| --- | --- | --- | --- | --- | --- | --- | --- | --- | --- | --- |
| 1 | Aston 2021 | To understand school psychologist’s views regarding the validity of cognitive tests with Black children. | Cross-sectional study | USA | School psychologist | Not provided | Jan, 2018 | 105 | Survey | Practice in intercultural encounters |
| 2 | Barba 2019 | To propose a consultation model that is a hybrid of multicultural and instructional consultation and includes use of the Cultural Assets Identifier to gather student strengths. | Case report | USA | School psychologist | Asset-based and multicultural school consultation | Not provided | 1 | Case description | Practice in intercultural encounters |
| 3 | Chu 2021 | To better understand how school psychologists perceive changes concerning student mobility in their contexts, including how they envision cross-cultural transitional care performed in their schools | Cross-sectional study | Lithuania | School psychologist | Third culture kids and Cross-cultural kids | Not provided | 200 | Survey | Practice in intercultural encounters |
| 4 | Edwin 2021 | To help school counselors and other MTSS interventionists assess culturally responsive practices for the purpose of designing MTSS interventions to adequately meet the academic and behavior needs of all students by accounting for the impact of culture. | Instrument validation study | USA | School psychologist | Cultural responsive practices | Not provided | 228 | Survey | Own cultural competence, sensitivity or similar |
| 5 | Golson 2022 | To evaluate the influence of student gender and race/ethnicity on school psychologists’ AU classification likelihood and confidence. | Mixed methods | USA | School psychologist | Not provided | Not provided | 229 | Survey | Own cultural competence, sensitivity or similar |
| 6 | Harris 2019 | To determine any differences in school psychologists’ perceived confidence across demographic variables related to conducting ASD assessment in schools generally and specifically with CLD students. | Cross-sectional study | USA | School psychologist | Not provided | Not provided | 148 | Survey with open-ended questions | Practice in intercultural encounters |
| 7 | Hurrell 2021 | To increase school nurse CC by developing a more formalized CC program that introduced the National CLAS Standards and followed with the development of education and resources customized to meet the needs of this specific group. | One-group pretest-posttest design with a convenience sampling | USA | School nurse | Not provided | During a school year | 18 | Survey, workshops, activity logs, feed-back | Own cultural competence, sensitivity or similar |
| 8 | Inkeroinen 2023 | To describe school nurses’ experiences of the health promotion they provide to school-age asylum seekers (6–17 years). | Qualitative research | Finland | School nurse | Not provided | Jan to Apr, 2019 | 12 | Interviews | Practice in intercultural encounters |
| 9 | Matza 2015 | To describe the results of the needs assessment and the actions taken to address the identified cultural competency needs for the U.S. school nurse workforce. | Cross-sectional study | USA | School nurse | Not provided | Fall 2012 | 2 645 | Survey | Own cultural competence, sensitivity or similar |
| 10 | Musliu 2019 | To describe school nurses’ experiences working with unaccompanied refugee children and adolescents. | Qualitative research | Sweden | School nurse | Trancultural nursing | Sept, 2017 to Jan, 2018 | 14 | Interviews | Practice in intercultural encounters |
| 11 | Parker 2019 | To replicate portions of Vieten et al.’s (2016) study among an exclusive sample of practicing school psychologists. | Cross-sectional study | USA | School psychologist | Not provided | Not provided | 121 | Survey | Own cultural competence, sensitivity or similar |
| 12 | Parker 2020 | To describe findings from interviews of school-based school psychologists who reported strategies for addressing cultural dynamics in consultation sessions. | Inquiry situated within the constructivist paradigm | USA | School psychologist | Multicultural school consultation framework | 2017 | 15 | Interviews | Practice in intercultural encounters |
| 13 | Reyna 2017 | To examine the validity of an existing measure of multicultural competence for use with school psychology practitioners. | Cross-sectional study | USA | School psychologist | Not provided | Not provided | 161 | Survey | Own cultural competence, sensitivity or similar |
| 14 | Sahamkhadam 2023 | To clarify the theoretical PCCDHS model and empirically test the assumptions of the interrelations between the constructs within the model. | Cross-sectional study | Sweden | School nurse | Process of Cultural Competence in the Delivery of Healthcare Services (PCCDHS) | August 2017 | 816 | Survey | Own cultural competence, sensitivity or similar |
| 15 | Sosa 2021 | To provide further empirical validation of the Nepantlera framework within the field of school social work as well as additional conceptual insights to advance it. | Qualitative research | USA | School social worker | Neplantlera | Not provided | 20 | Interviews | Practice in intercultural encounters |
| 16 | Suk 2015 | To examine the current status of multicultural attitudes and cultural sensitivity of school nurses in Korean elementary schools and investigate the mediating effect of their self-efficacy as school nurses on the relationships between multicultural attitudes and cultural sensitivity. | Cross-sectional study | Korea | School nurse | Cultural sensitivity | July to Dec, 2013 | 157 | Survey | Own cultural competence, sensitivity or similar |
| 17 | Teasley 2014 | To directly follow up with similar survey research methods used in Teasley’s (2005) exploratory study on perceived levels of cultural competence among school social workers. | Cross-sectional study | USA | School social worker | Not provided | October 2008 | 285 | Survey | Own cultural competence, sensitivity or similar |
| 18 | VelascoLeon 2020 | To explore the most common practices of school psychologists and counsellors in Australia in the assessment of CALD students who present with academic difficulties and compare these practices to what the literature suggests are quality assessment practices. | Cross-sectional study | Australia | School psychologist | Not provided | 7 months | 34 | Survey | Practice in intercultural encounters |
| 19 | Wahlström 2021 | To investigate school nurses’ descriptions of promoting participation for children of foreign origin in health visits. | Cross-sectional study | Sweden | School nurse | Not provided | Aug, 2017 to Jan 2018 | 673 | Survey with open-ended questions | Practice in intercultural encounters |
| 20 | Wahlstrom 2020 | To investigate associations between school nurses' self-assessed cultural competence in health visits with children of foreign origin and demographic variables. | Cross-sectional study | Sweden | School nurse | Process of Cultural Competence in the Delivery of Healthcare Services (PCCDHS) | Aug, 2017 to Jan 2018 | 816 | Survey | Own cultural competence, sensitivity or similar |
| 21 | Aganza 2015 | To describe the assessment and use of cultural assets in records review, interview, observation, testing, and intervention development in the context of an ecosystemic approach. | Case study | USA | School psychologist | Cultural assets | Not provided | 4 | Case study | Practice in intercultural encounters |
| 22 | Leinander 2019 | To describe first school health dialogue meetings with newly arrived child migrants and their families from the perspective of primary school nurses. | Qualitative interview study | Sweden | School nurse | Not provided | End of 2017 to early 2018 | 7 | Semi-structured interviews | Practice in intercultural encounters |
| 23 | Sakata 2024 | To address gaps in EP research by answering the main research question: How can EPs develop culturally responsive practice (CRP)? | Delphi method | UK | School psychologist | Culture and culturally responsive practice | Not provided | 23 | Systematic literature review and survey | Practice in intercultural encounters |
