# Supplementary material for: School health professionals’ understanding of culture: a scoping review
Source: BMJ Open. 2025 Jul 25;15(7):e100689. doi: 10.1136/bmjopen-2025-100689 (PMC12306337; doi:10.1136/bmjopen-2025-100689)
Supplement: Supplementary file 4 [file bmjopen-2025-100689.reviewer_comments.pdf]

PEER REVIEW HISTORY

BMJ Open publishes all reviews undertaken for accepted manuscripts. Reviewers are asked to complete a checklist review form and are provided with free text boxes to elaborate on their assessment. These free text comments are reproduced below.

ARTICLE DETAILS

Title (Provisional)

School health professionals’ understanding of culture – a scoping review

Authors

Wahlström, Emmie; Landerdahl Stridsberg, Sara; Larsson, Camilla; Stier, Jonas

---

VERSION 1 - REVIEW

**Reviewer**	**1**
**Name**	**Eaton, Andrew**
**Affiliation**	**University of Regina, Faculty of Social Work - Saskatoon Campus**
**Date**	**07-Apr-2025**
**COI**	**None**

This scoping review presents a content analysis of school health professionals' (primary psychologists and nurses) understanding of culture. Overall, I found the Results and Discussion compelling and that the review was conducted transparently. I'm curious as to why cultural competency, sensitivity, and/or humility were largely not discussed (brief mention is made) and I detail this and other issues below.

Title

- "Exploring" suggests the authors conducted primary research, but this is a synthesis. I recommend "School health professionals' understanding of culture: a scoping review".

Abstract

- The Introduction should immediately address school health professionals, as this synthesis does not pertain to all health professionals.

- Design should specify "school health professionals'"

- Analysis should have a bit more detail on the qualitative content analysis. If space is needed, it is not necessary to list all databases in the abstract (can just say "10 databases were searched").

Strengths and limitations

- This section is supposed to be article highlights, per journal instructions. Yet the final point states four professions synthesized when the scoping review really only speaks to two (school psychologists and school nurses).

- Please include limitations in a header that is titled "Strengths and limitations of this study".

Introduction

- The first paragraph makes numerous uncited claims.

- I recommend against employing so many uncommon acronyms. SHS is okay - it's uncommon but used frequently - but each profession can be spelled out at each mention.

- It is a gap that an article focused on practice does not review key concepts such as "cultural competency", "cultural sensitivity", and/or "cultural humility" - particularly as these are a part of curricula for many people in these professions.

Methods

- Was the review pre-registered?

- Please justify the cutoff date for included articles.

- Was "culture" the only term that would warrant inclusion? Many Western studies on this topic would use "race" or "ethnicity". Were these terms also included? If not, this is a major limitation.

- "Characteristics of the literature" would be more clearly presented in a table.

Limitations

- Was language a limitation (e.g., did included articles have to be in English)?

General

- The citation style does not align with the Journal.

- A copyedit is needed. There are key words missing from sentences, incorrect tenses, and other issues.

**Reviewer**	**2**
**Name**	**Kayzuka, Cezar**
**Affiliation**	**University of Sao Paulo, Pharmacology**
**Date**	**15-Apr-2025**
**COI**	**None**

Thank you for the opportunity to review this manuscript. The manuscript addresses the understanding of culture by healthcare professionals in the school setting. Overall, the

manuscript’s design and description are appropriate and address relevant topics to improve culturally sensitive healthcare delivery in schools.

My suggestions to improve the study description are:

1 - Study purpose and review questions -

The study objective is appropriate and clearly stated. However, the five review questions currently presented may benefit from some refinement. While the authors provide a structured general objective, the overarching review question based on the PCC framework is not explicitly stated. As a result, some of the questions appear somewhat repetitive. It might be helpful to consolidate these questions, as only questions two and three seem to add unique elements beyond the general aim. Question four, which refers to the geographic context, is usually implicit in the nature of scoping reviews and may not require separate mention.

2 - Methods - Screening process -

Although the authors provide a clear description of the screening process, there is no reference to the inter-rater reliability score, which is only mentioned later as a limitation of the study due to its moderate result. As a native tool of Covidence used by the authors, providing this result is important for understanding and supporting the reliability of the screening process.

3 - Results -

The description of the included studies’ characteristics, participants, concept, and context is too extensive, which may make it difficult for readers to identify the main findings. As this information is already presented in Supplementary File 3, a more concise summary focused on the key findings relevant to the review objectives may improve clarity. Additionally, the extensive use of in-text citations interrupts the flow of the results section and may be unnecessary, given that references are already listed in SF2 and SF3 (PRISMA-ScR). Consider using visual aids such as figures, tables, or graphs to support data presentation more effectively.

4 - Limitations -

Limitation number one is appropriate, but lacks supporting data and could be strengthened by including the actual inter-rater reliability score in the methods section.

Regarding the second limitation, it may not be necessary to describe it here, as the excluded studies could simply be mentioned in the screening results (e.g., "two studies were excluded later due to misalignment with the study focus").

Finally, the discussion on the authors’ cultural backgrounds and potential biases may be better placed at the end of the methods section, as it serves more as a transparency statement than a methodological limitation.

VERSION 1 - AUTHOR RESPONSE

Reviewer 1 comments	Response by authors
This scoping review presents a content analysis of school health professionals' (primary psychologists and nurses) understanding of culture. Overall, I found the Results and Discussion compelling and that the review was conducted transparently. I'm curious as to why cultural competency, sensitivity, and/or humility were largely not discussed (brief mention is made) and I detail this and other issues below.	Thank you. We address your concern regarding the concepts and issues below.
Title<br>- "Exploring" suggests the authors conducted primary research, but this is a synthesis. I recommend "School health professionals' understanding of culture: a scoping review".	We have adjusted accordingly.
Abstract<br>1) The Introduction should immediately address school health professionals, as this synthesis does not pertain to all health professionals.<br>2) Design should specify "school health professionals'"<br>3) Analysis should have a bit more detail on the qualitative content analysis. If space is needed, it is not necessary to list all databases in the abstract (can just say "10 databases were searched").	Abstract<br>1) We have adjusted accordingly.<br>2) We have adjusted accordingly.<br>3) We have added a sentence describing which data was analyzed using qualitative content analysis. We have also removed the names of the databases and replaced this with "in ten databases" as suggested.
Strengths and limitations<br>1) This section is supposed to be article highlights, per journal instructions. Yet the final point states four professions synthesized when the scoping review really only speaks to two (school psychologists and school nurses).<br>2) Please include limitations in a header that is titled "Strengths and limitations of this study".	1) We have removed the bullet point stating the professions.<br>2) We have included two new bullet points stating limitations of the study below the heading "Strengths and limitations of this study".
Introduction<br>1) The first paragraph makes numerous uncited claims.<br>2) I recommend against employing so	1) Citations of references have been added to the two sentences that did not have a citation. (line 4, page 4 and line 9, page 4).

many uncommon acronyms. SHS is okay - it's uncommon but used frequently - but each profession can be spelled out at each mention.<br>3) It is a gap that an article focused on practice does not review key concepts such as "cultural competency", "cultural sensitivity", and/or "cultural humility" - particularly as these are a part of curricula for many people in these professions.	2) All acronyms for the professions have been removed and the professions are spelled out.<br>3) Thank you for this reflection and we agree that concepts such as cultural competence or sensitivity are often included in education of health professionals. Still, the concept in focus of the review is *culture* – not the uncountable word combination concepts. We have provided a brief overview of the concept of culture on page 5-6. We have chosen culture as we have found, through our previous research and overview of the field, that concepts embedding culture by surrounding it with a prefix or a word combination does not always define what culture is. We also chose culture to capture all possible variations of how culture is embedded and combined as opposed to narrowing the scope to specific culture-related concepts. To provide some clarification on this, we have included a statement on page 9 saying that "any word combination where culture is included was included as a search term". We assumed that conducting searches on culture without narrowing to a specific culture-related concept would generate the most thorough searches of the field.<br><br>In addition, it could be noted that the focus on practice in the articles was part of the results, not the aim of the conducted searches. These and related results have also been discussed in the discussion section. There, we have discussed the conceptualization of culture in the articles as well as the lack of research on intercultural encounters albeit the numerous frameworks, models and concepts describing how a cultural competent, sensitive, responsive, etc care may be provided.
Methods<br>1) Was the review pre-registered?<br>2) Please justify the cutoff date for included articles.<br>3) Was "culture" the only term that would warrant inclusion? Many Western studies on this topic would use "race" or "ethnicity". Were these terms also	1) Yes, we have published a scoping review protocol. This protocol is cited in Methods on page 7 and in the list of references.<br>2) The justification of the cut-off date has been expanded, see page 8, second paragraph.<br>3) Yes, culture was the only term that warranted inclusion. We chose to solely focus on culture as this is a broad concept

included? If not, this is a major limitation.<br>4) "Characteristics of the literature" would be more clearly presented in a table.	in itself. Adding concepts such as "race" and "ethnicity" would have resulted in another interesting review that may very well be needed. However, we do not concur with any underlying assumption that there is a "natural" link between culture, race and ethnicity. There is a tendency that people (professionals and researchers) sometimes use culture as a signifier indicating difference from a national majority population, often when speaking of migrants. Thus, it is reduced to a fixed characteristic "attributed to "certain homogeneous groups" based on cultural characteristics, ethnicity, language, beliefs, values, traditions, etc. (Stier, 2009; Zanting et al., 2024)", as described on page 6. We would argue against this kind of conceptual understanding of culture and rather rely on definitions by Stuart Hall and others stating that culture is a process and a set of practices shared by a group of people or a society for interpreting and making sense of the world around them.<br>4) Thank you for this suggestion. As reviewer 2 also highlighted this issue we have chosen to shorten the text to convey the key points and added a reference to supplement 3, where a more detailed information on each article is provided. We considered adding a table but realized that there would be a considerable overlap between such a table and supplement 3. Hopefully these revisions have made the text more accessible.
Limitations<br>- Was language a limitation (e.g., did included articles have to be in English)?	- Yes, language was a limitation given that articles had to be published in Danish, English, German, Norwegian, or Swedish. We have added this as a limitation on page 27 in the section Limitations.
General<br>- The citation style does not align with the Journal.<br>- A copyedit is needed. There are key words missing from sentences, incorrect tenses, and other issues.	1) Thank you for highlighting this. We have adjusted the citation style according to the journal's guidelines.<br>2) Prior to submitting the manuscript to the journal, it was language proofread by a professional language proofreading service. It would thus be helpful if we could get some clearer directions as to which parts of the manuscript that need further copyedit so that we might report these shortcomings to the services used and address the specific parts. Prior to resubmitting the manuscript, we have

	read through the text thoroughly and revised when needed.

**Reviewer 2 comments**	**Response by authors**
Comments to the Author:<br>Thank you for the opportunity to review this manuscript. The manuscript addresses the understanding of culture by healthcare professionals in the school setting. Overall, the manuscript's design and description are appropriate and address relevant topics to improve culturally sensitive healthcare delivery in schools.	Thank you.
1 - Study purpose and review questions - The study objective is appropriate and clearly stated. However, the five review questions currently presented may benefit from some refinement. While the authors provide a structured general objective, the overarching review question based on the PCC framework is not explicitly stated. As a result, some of the questions appear somewhat repetitive. It might be helpful to consolidate these questions, as only questions two and three seem to add unique elements beyond the general aim. Question four, which refers to the geographic context, is usually implicit in the nature of scoping reviews and may not require separate mention.	As suggested, we have revised the review questions and removed number 4 and 5. We agree that this provides more clarity. We have chosen to keep question no 1 as this, although formulated similar to the review aim, provides another dimension (directed to the concept as such) than cultural self-understanding and intercultural interaction and communication.
2 - Methods - Screening process - Although the authors provide a clear description of the screening process, there is no reference to the inter-rater reliability score, which is only mentioned later as a limitation of the study due to its moderate result. As a native tool of Covidence used by the authors, providing this result is important for understanding and supporting the reliability of the screening process.	Text on inter-rater reliability has been added to the methods section (p.11) and in the bullet point limitations (after the abstract). The text on inter-rater reliability in the limitations has also been revised.
3 - Results -<br>The description of the included studies' characteristics, participants, concept,	Thank you for this suggestion. As reviewer 1 also highlighted this issue we have chosen to shorten the text to convey the key points and added a reference to supplement 3, where more

and context is too extensive, which may make it difficult for readers to identify the main findings. As this information is already presented in Supplementary File 3, a more concise summary focused on the key findings relevant to the review objectives may improve clarity. Additionally, the extensive use of in-text citations interrupts the flow of the results section and may be unnecessary, given that references are already listed in SF2 and SF3 (PRISMA-ScR). Consider using visual aids such as figures, tables, or graphs to support data presentation more effectively.	detailed information on each article is provided. We considered adding a table but realized that there would be a considerable overlap between such a table and supplement 3. We have also changed the citation style to fit with the Journal guidelines, thus addressing the comment about in-text citations. Hopefully these revisions have made the text more accessible.
4 - Limitations -<br>1) Limitation number one is appropriate, but lacks supporting data and could be strengthened by including the actual inter-rater reliability score in the methods section.<br>2) Regarding the second limitation, it may not be necessary to describe it here, as the excluded studies could simply be mentioned in the screening results (e.g., "two studies were excluded later due to misalignment with the study focus").<br>3) Finally, the discussion on the authors' cultural backgrounds and potential biases may be better placed at the end of the methods section, as it serves more as a transparency statement than a methodological limitation.	1) As previously mentioned, we have added text on inter-rater reliability to the methods section (p.11), in the bullet point limitations (after the abstract) and revised the text in the limitations section.<br>2) We have adjusted accordingly, see revised text on page 11.<br>3) We have adjusted accordingly and placed the text below the heading "reflexivity" at the end of the methods section.

VERSION 2 - REVIEW

**Reviewer**	**1**
**Name**	**Eaton, Andrew**
**Affiliation**	**University of Regina, Faculty of Social Work - Saskatoon Campus**
**Date**	**01-Jul-2025**

COI

The authors have addressed my prior concerns and the manuscript is improved.

**Reviewer**	**2**
**Name**	**Kayzuka, Cezar**
**Affiliation**	**University of Sao Paulo, Pharmacology**
**Date**	**30-Jun-2025**
**COI**	

I have reviewed the revised version of the manuscript, and I am pleased to confirm that the authors have adequately addressed all my previous comments and suggestions. The revisions have significantly improved the quality of the paper, and I now fully support its publication in its current form.

Thank you for the opportunity to evaluate this work.
